# Beriberi

**Published:** 1902-09-27

**Authors:** 


### BERIBERI.

The discussion on beriberi which took place at the Manchester meeting of the British Medical Association, was remarkable for the absolute divergence of the views expressed by the various speakers, and was thus fairly representative of professional opinion as it exists at the present time in regard to this very important disease. Dr. Patrick Manson pointed out the probability that the term beriberi is not infrequently applied to diseases which are not of that nature, and said that until we have discovered the *vera causa* of the disease, and until we know how to recognise this in our cases, we are sure to be more or less at sea in our definitions, descriptions and diagnoses. Undoubtedly there are in the tropics cases of peripheral neuritis arising from other causes than beriberi, and such cases when occurring in beriberi districts must often be taken for instances of that disease. He held, however, that the form of neuritis which is known as beriberi is produced by a toxin, the product of a germ operating in some culture medium located outside the body; that the said toxin enters the body neither in food nor in water; and that one must therefore conclude that it is either introduced through the skin or is inhaled. The proof that the toxin of beriberi is produced by a living germ lies in the circumstance that the disease can be introduced into virgin country and there spread. Spontaneous multiplication is a property peculiar to living things, therefore the originating agent of beriberi is a living thing, a germ. On the other hand, it is not in man's body that it grows, for it is now well recognised that the most important measure in the management of a case of beriberi is the removal of the patient from the place in which he sickened. If removed in time he will almost invariably recover. Dr. Manson therefore argues that the cause of beriberi cannot be a germ living and multiplying in the body of the patient, for if that were the case the patient, when he left the endemic spot, would still carry the germ with him and the disease it produces would continue until immunity had been produced. The culture medium, therefore, must be something outside the human body, and, in illustration of the fact, Dr. Manson draws an analogy between beriberi and alcoholism. Alcohol, the toxin which gives rise to alcoholic neuritis, is produced during the proliferation of a germ—the yeast plant—in a saccharine solution. We may swallow the germ with impunity, and may swallow the culture medium—the saccharine solution—even with benefit, but the product of the operation of the germ on the culture medium — alcohol — the toxin produced outside the body, is a poison. That the toxin of beriberi does not enter the body in either food or water is argued from a series of instances in which different groups of people were differently affected in regard to the disease although apparently taking the same food and supplied with water from the same source. Captain Rost, I.M.S., however, took a very different view, maintaining that there is an intimate connection between a certain disease in rice and beriberi in man, and gave many facts to prove his case. Dr. Sambon also held to the view that rice might be related to beriberi in much the same way as we now believe pellagra to be related to maize, or in other words that rice may become a vehicle of the beriberi infection. He not only doubted some of the propositions put forward by Dr. Manson, but also questioned the analogy with alcoholic neuritis which he had employed as an illustration. Moreover he believed that the specific agent of beriberi lives within the patient's body and attacks the peripheral nerves, which was quite opposed to what Dr. Manson had maintained. Yet he did not believe in direct infection from man to man, except in the presence of its peculiar agent of propagation, whatever that may turn out to be. Evidently there is much to learn concerning this strange and fatal disease.

